# The Role of LncRNAs in Uveal Melanoma

**DOI:** 10.3390/cancers13164041

**Published:** 2021-08-11

**Authors:** Paula Milán-Rois, Anan Quan, Frank J. Slack, Álvaro Somoza

**Affiliations:** 1 Instituto Madrileño de Estudios Avanzados en Nanociencia (IMDEA Nanociencia), Unidad Asociada al Centro Nacional de Biotecnología (CSIC), 28049 Madrid, Spain; paula.milan@imdea.org; 2 Department of Pathology, Beth Israel Deaconess Medical Center (BIDMC)/Harvard Medical School, Boston, MA 02215, USA; aquan@bidmc.harvard.edu (A.Q.); fslack@bidmc.harvard.edu (F.J.S.)

**Keywords:** lncRNA, uveal melanoma, cancer, noncoding RNA, epigenetics, therapy, diagnosis

## Abstract

**Simple Summary:**

Uveal melanoma is a rare cancer with a bad prognosis that needs new therapeutic and diagnostic approaches. In this regard, long non-coding RNAs (lncRNAs) play a pivotal role in cancer, among other diseases, and could be used as therapeutic targets of diagnostic markers. In this review, lncRNAs related to uveal melanoma are revealed to better understand their mechanism of action, and inspire the development of novel treatment and diagnostic approaches. In addition, the interaction of lncRNA with other non-coding RNAs (ncRNAs) is also discussed since it might be one of the most relevant mechanisms of action. The compiled information is helpful not only for uveal melanoma experts, but also for ncRNA cancer researchers.

**Abstract:**

Uveal melanoma (UM) is an intraocular cancer tumor with high metastatic risk. It is considered a rare disease, but 90% of affected patients die within 15 years. Non-coding elements (ncRNAs) such as long non-coding RNAs (lncRNAs) have a crucial role in cellular homeostasis maintenance, taking part in many critical cellular pathways. Their deregulation, therefore, contributes to the induction of cancer and neurodegenerative and metabolic diseases. In cancer, lncRNAs are implicated in apoptosis evasion, proliferation, invasion, drug resistance, and other roles because they affect tumor suppressor genes and oncogenes. For these reasons, lncRNAs are promising targets in personalized medicine and can be used as biomarkers for diseases including UM.

## 1. Introduction

For decades, a significant volume of research has been devoted to unraveling genes that encode proteins. However, in recent years, the non-coding genome has revolutionized biology, with more than 90% of the RNA in the human genome consisting of non-coding RNAs (ncRNAs). Furthermore, it has been demonstrated that ncRNAs have an essential role in cellular processes involving homeostasis and disease progression [1]. The complex network of interactions between multiple ncRNAs as well as between ncRNAs and coding RNAs highlights the ncRNAs’ fundamental role in regulating cellular processes, meaning that ncRNAs are taking part in several points of the cellular pathways controlling the expression of key genes. Therefore, dysregulation of ncRNA is directly related to neurodegenerative, developmental, metabolic diseases, or cancer.

The different types of ncRNAs can be classified by size into two main groups. Those with a length below 200 nucleotides (nts) are known as small ncRNAs such as the microRNAs (miRNAs), t-RNA-derived small RNAs (tsRNAs), and PIWI-interacting RNAs (piRNAs). In contrast, long non-coding RNAs (lncRNAs) include those above 200 nts in length including circular RNAs (circRNAs) and pseudogenes [1].

Although signaling pathways have long been characterized, lncRNAs are understudied, however, they have been shown to play an unexpected and essential role. Recently, lncRNAs have been discovered as critical players in regulatory networks. They interact with signaling molecules and regulators by making them more flexible or open to changes in the environment [2]. LncRNAs are involved in cellular pathways and mechanisms such as stem cell pluripotency, cell cycle regulation, metabolism, aging, cancer, and neurodegenerative and cardiovascular diseases [3,4]. They have a critical effect on the proliferation, invasion, or metastasis of tumors [2], which can be exploited to develop therapeutic agents or allow specific lncRNAs to be used as biomarkers [5].

However, substantial work is needed to understand the role of lncRNAs in homeostasis and disease progression. Indeed, it is rather challenging to elucidate the lncRNA roles and their implications in genetic regulation such as their effects in chromosome domain organization, nucleic acids and transcription factors subcellular localization, expression patterns, and genetic evolution or stability [6]. For example, lncRNAs are poorly conserved, which may seem contradictory with their relevant role in the cells. Furthermore, some lncRNAs can encode small functional peptides, suggesting that lncRNAs could also function as coding sequences [7]. Moreover, their flexibility and relatively large size have made it quite difficult to resolve their structures by classical methods such as X-ray crystallography, NMR spectroscopy, or electron microscopy. Moreover, lncRNAs play so many roles that it is complicated to assign roles to each annotated lncRNA [8]. LncRNA role assignation is done by loss of function approaches such as RNAi, ASOs, and CRISPR techniques. However, the inhibition by these processes is not as effective as in the case of mRNA, which complicates the elucidation of their roles [6]. Although these factors make it more complicated to unravel the biological function of lncRNAs, their roles are being studied individually and grouped according to their interaction with other molecules in cellular pathways [4].

In this review, we focus on long non coding-RNAs due to their crucial role in gene regulation through their interaction with proteins, DNA, and RNA. In particular, it is worth highlighting their interaction with a specific class of ncRNAs, microRNAs, as both have been implicated as genome master regulators [6].

**Figure 1:**
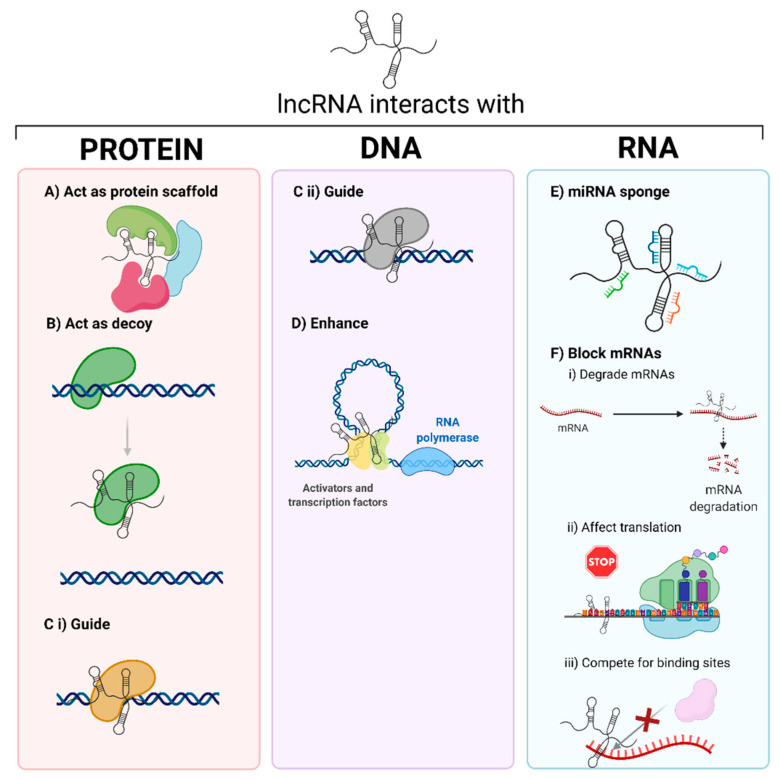
The interaction of lncRNAs with different biomolecules tunes their biological activity. LncRNAs can interact with proteins, DNA, or RNA, acting as (**A**) scaffolds of proteins, (**B**) decoys, preventing protein–oligonucleotide complex formation, (**C**) guides for (**i**) proteins or (**ii**) oligonucleotides, (**D**) enhancers, promoting transcription, (**E**) miRNAs sponges, or (**F**) mRNA inhibitors, blocking mRNA function due to (**i**) mRNA degradation, (**ii**) blocking translation, or (**iii**) competing for binding sites. Created with BioRender.com, accessed on 26 October 2020.

Due to the variety of molecules with which lncRNAs can interact, their mechanism of action and functions are highly diverse (Figure 1). The biological processes in which lncRNA take part are included in Table 1 [4,8]. Their mechanism of action and function vary depending on the interaction molecule. Significant efforts have been dedicated to elucidate new lncRNA interactions and mechanisms. To achieve this goal, both experimental and computational techniques are essential to investigate new candidates and relate them to various diseases [2,3,4,9].

LncRNA–protein interactions are involved in transcription, post-transcription, splicing, molecular scaffolds, or decoys. For example, these interactions can allow for protein-DNA recognition to induce or repress transcription, the recruitment of chromatin-modifying enzymes, or the cooperation with splicing factors (involved in the regulation of alternative splicing). The current methods to detect these interactions are electrophoresis, RNA-pull down assay, fluorescence in-situ hybridization (FISH) colocalization, oligonucleotide-targeted RNase H assay, and high throughput transcriptomics or proteomics [2,24].

LncRNA interactions with DNA have been implicated in transcription (e.g., participating in enhancers or chromatin looping processes), DNA repair capacity, and nuclear body formation and function. The techniques to elucidate these mechanisms are based on chromatin isolation by RNA purification (ChIRP), capture hybridization analysis of RNA targets (CHART), RNA antisense purification (RAP), chromatin oligo affinity precipitation (ChOP) [24,25,26], and recently computer tools such as GRIDseq, Triplexator, or LongTarget [27].

LncRNAs can also interact with ncRNAs such as microRNAs, which leads to their inhibition or activation. Additionally, they can interact with mRNAs, affecting their alternative splicing or stability, inhibiting translation, or even competing for binding sites [25]. Some techniques to study these interactions include selective 2′-hydroxyl acylation analyzed by primer extension sequencing (SHAPE-Seq), RNA antisense purification (RAP), selective 2′-hydroxyl acylation analyzed by primer extension mutational profiling (SHAPE-Map), dimethyl sulfate sequencing (DMS-Seq), fragmentation sequencing (FRAG-seq), parallel analysis of RNA structure (PARS), parallel analysis of RNA structure with temperature elevation (PARTE) or in vivo click selective 2′-hydroxyl acylation analyzed by primer extension (icSHAPE) [25].

LncRNAs can be classified according to their *cis* or *trans* function or position relative to coding genes [2,28], the latter of which is the preferred method [2,29]. This classification categorizes lncRNA into the following types:

- Intergenic, the lncRNAs transcribed from DNA strands between protein-coding genes. These lncRNAs act as master regulators of transcription and posttranscriptional and translation processes [30].
- Intronic, the lncRNAs transcribed from introns in the same orientation as the mRNA of protein-coding genes. Many of these lncRNAs are implicated in alternative splicing [31].
- Overlapping, the lncRNAs transcribed from overlapping mRNA of protein-coding genes. Many overlapping lncRNAs have implications in splicing, tissue specificity, and aging [32].
- Antisense, the lncRNAs transcribed from the opposite strand (antisense) of protein-coding genes. These lncRNAs can interfere with transcription or mRNA stability [33].

Some members of all of these types are related to diseases; for example, there are intergenic lncRNAs such as Doublesex And Mab-3 Related Transcription Factor 2 (DMRT2), involved in obesity progression, and linc1992, involved in immune disorders [30]; intronic lncRNAs such as Prostate Cancer Associated Transcript 19 (PCAT19) associated with poor prostate cancer prognosis [34]; overlapping lncRNA Sex determining Region Y-box 2 (SOX2) promotes Ewing’s sarcoma proliferation [35]; antisense lncRNAs such as HOXA-AS2 promotes many human cancers [36] or lncRNA β-secretase 1 antisense (BACE1-AS) related to Alzheimer’s disease [8]. lncRNAs associations with multiple diseases indicate that they might be considered as targets for therapeutic or diagnostic system development [2,37].

LncRNAs are involved in many of the hallmarks of cancer as described by Hanahan and Weinberg [38] such as proliferation, motility, immortality, angiogenesis, inflammation, drug resistance, genomic stability, and cell viability. In fact, recent studies of transcriptome profiles generated via next-generation sequencing have found many lncRNAs to be mutated or abnormally expressed in tumors [39]. In this sense, lncRNAs could drive cancer phenotypes, acting as tumor suppressors, oncogenes, or both [40,41]. It is necessary to correlate the activity of each lncRNA with key cancer players to determine which lncRNA belongs in which category [42,43]. The cancer lncRNA list is continuously growing and compiled in databases such as Lnc2Cancer or the Cancer LncRNA Census [8,44,45]

**Figure 2:**
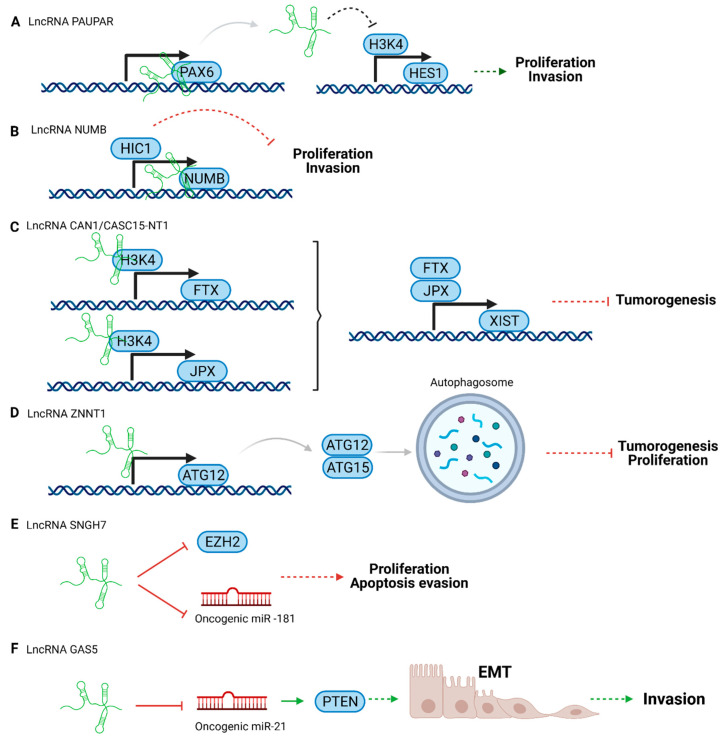
Scheme of tumor suppressor lncRNA pathways in uveal melanoma. (**A**) lncRNA PAUPAR, (**B**) lncRNA NUMB, (**C**) lncRNA CAN1/CASC15-NT1, (**D**) lncRNA ZNNT1, (**E**) lncRNA SNGH7, and (**F**) LncRNA GAS5. Created with BioRender.com, accessed on 26 October 2020.

In healthy cells, tumor suppressor genes are activated when cells detect oncogenic stress to maintain homeostasis. Interestingly, several lncRNAs are involved in regulating those genes [42], leading mainly to reducing tumor cell growth, proliferation, invasion, and metastasis. However, when these genes or lncRNAs are downregulated, cancer develops [44]. Tumor suppressor lncRNAs (Figure 2) can be used as therapeutic agents because they can interact with oncogenes, directly or indirectly, reducing their expression levels and, therefore, tumor progression [42].

**Figure 3:**
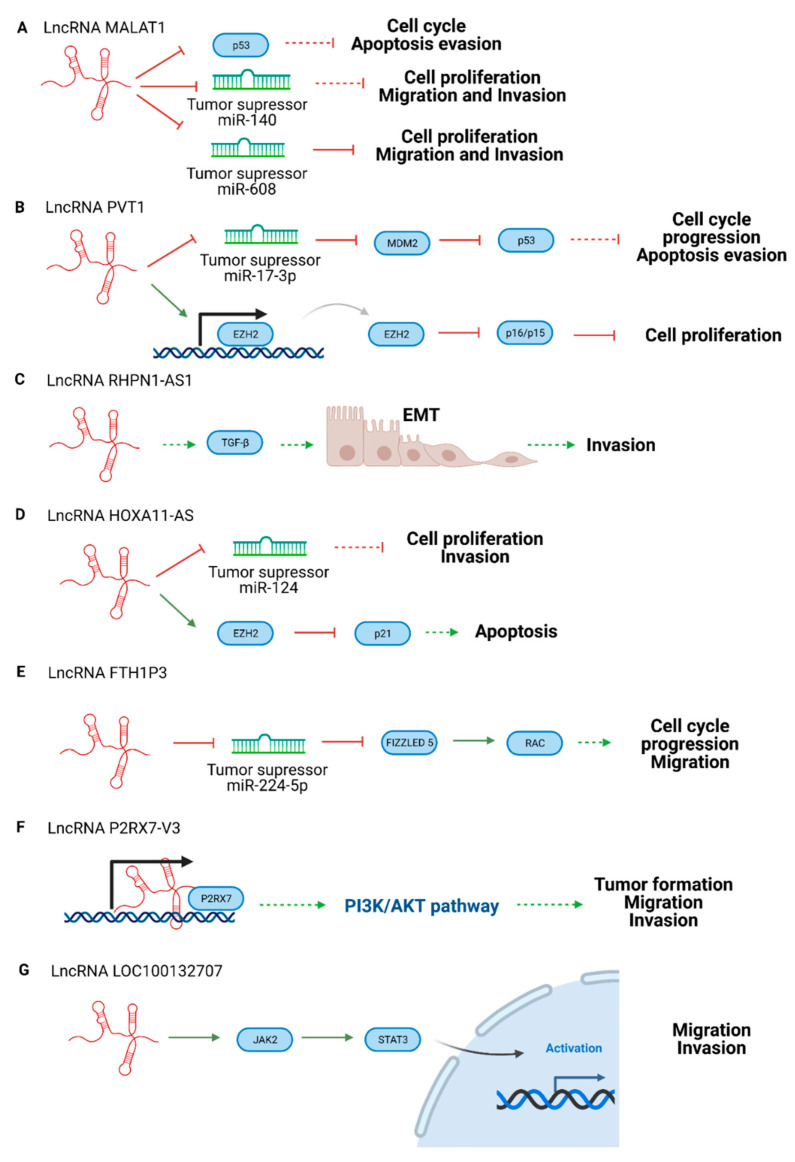
Scheme of oncogenic lncRNA pathways in UM. (**A**) LncRNA MALAT1, (**B**) lncRNA PVT, (**C**) lncRNA RHPN1-AS1, (**D**) lncRNA HOXA11-AS, (**E**) lncRNA FTH1P3, (**F**) LncRNA P2RX7-V3, and (**G**) LncRNA LOC100132707. Created with BioRender.com.

In addition, there are homeostatic pathways involved in cellular proliferation, for example, gastric epithelial maintenance. When these processes are deregulated, cancer appears. Oncogenic lncRNAs (Figure 3) play pivotal roles in oncogenic transformation because they are involved in cellular pathways that promote carcinogenesis [42,45]. Oncogenic lncRNAs are usually upregulated in cancer and promote cell growth, angiogenesis, migration, invasion, apoptosis evasion, and chemoresistance [46]. Oncogenic lncRNAs are promising therapeutic targets because they can be inhibited by siRNAs, antisense oligonucleotides (ASOs), or via Clustered Regularly Interspaced Short Palindromic Repeats (CRISPR) techniques, leading to tumorigenesis reduction [47]. In addition, they can be used as biomarkers because high levels of these lncRNAs can be related to tumor surveillance [42].

LncRNAs have been found to be involved in many types of cancers such as prostate, breast, brain, lung, liver, pancreatic, colorectal, renal, ovarian, and gastric cancer [2,48]. However, lncRNA responses to cancer treatments (e.g., chemotherapy or immunotherapy) or their relationship with the tumor microenvironment are understudied [8].

In this review, we have described the roles of these ncRNAs in a rare type of cancer of the eye, uveal melanoma (UM).

## 2. Uveal Melanoma

Uveal melanoma (UM) is a type of intraocular cancer tumor. It is considered a rare disease due to its low prevalence, making up just 3–5% of all cancers within the U.S. population [49]. It principally appears in the choroid, iris, and ciliary body. UM has a high metastatic risk and a poor prognosis as 90% of patients die within 15 years of diagnosis [50].

In most (83%) cases, UM is caused by mutations in the alpha subunit of the heterotrimeric G gene (GNAQ) or its paralogue GNA11. In addition, UM usually presents other genetic alterations such as the loss in chromosome 3 and gain in chromosome 8q. The prognosis of this disease is worrying since patients frequently undergo liver metastasis and succumb within 2–6 months [51]. In metastatic UM, there are also mutations in breast cancer gene (BRCA) associated protein 1 gene (BAP1) on chromosome 3, which results in aggressive cancer [52].

Besides the genetic causes, there are also critical epigenetic alterations in the UM carcinogenesis process (e.g., ncRNA abnormalities, DNA methylation, and histone modifications) [53,54,55] that are also understudied. Regarding ncRNAs, it is described that microRNAs and lncRNA dysregulated levels affect the tumorigenesis process and the final prognosis. LncRNAs affect several points of the UM pathways, notably at the MAPK/ERK or PI3K/AKT pathways [56]. These aberrations should be taken into account with the alterations described by Thornton et al. to classify the patients in proper treatments groups [55].

Enucleation, radiotherapy, or laser therapy are the current standard therapeutic options for primary resectable UM tumors. However, for unresectable and/or metastatic tumors, systemic chemotherapy is the main treatment. Since UM cells are drug-resistant in primary and metastatic tumors, chemotherapy is usually applied as a combination of different drugs such as dacarbazine, temozolomide, gemcitabine, or treosulfan [57]. Nonetheless, in a meta-analysis of current treatments (e.g., chemotherapy or radiotherapy) for metastatic UM, all treatments positively affected overall survival or reduced the metastatic risk [58,59,60]. However, there are promising novel treatments in clinical trials that combine chemotherapy agents with photodynamic therapy, immunotherapy, or targeted therapy for metastatic UM [61]. Despite this progress, the aberrations compilation indicates UM is a disease with a poor prognosis and low survival rates. For these reasons, new treatments and diagnosis methods are needed not only to focus on genetic, but also epigenetic drivers.

## 3. LncRNAs in Uveal Melanoma

As above-mentioned, dysregulated lncRNAs are implicated in many cancers including UM. In the following sections, their role in UM is discussed, and their main mechanisms and roles are summarized in Table 2 [62].

Many of the lncRNAs compiled in this article are those broadly studied in other cancers. Nevertheless, less lncRNA research has been conducted in UM, so it needs further investigation in the UM field. In some cases, the mechanism involved in UM is not the same as the one previously studied, and oncogenic lncRNAs act in UM as tumor suppressors or vice versa.

### 3.1. Tumor Suppressor LncRNAs in Uveal Melanoma

Tumor suppressor lncRNAs can act directly on effector molecules or act as regulator elements, for instance, controlling transcription. Depending on the mechanism of action, they can activate tumor suppressor pathways (Figure 2B–D) or block the tumorigenesis process (Figure 2A,E).

Some examples of tumor suppressors in UM are presented below (Figure 2).

LncRNA Pax6 Upstream Antisense RNA (PAUPAR) is a lncRNA that is transcribed upstream of the PAX6 transcription factor. It was first identified in neuroblastoma modulating Paired Box 6 (PAX6) activity [79]. This protein controls progenitor cell potency and proliferation, specification, and spatial patterning in neural cells [80,81]. LncRNA PAUPAR is downregulated in UM, blocking cell migration and tumor formation. Its downstream target is Hairy and Enhancer of Split 1 (HES1), a critical player in the NOTCH signaling pathway, controlling the survival or apoptosis of melanocytes [73]. Highly expressed in UM, HES1 promotes proliferation and invasion [82]. Increased levels of lncRNA PAUPAR alters the expression of HES1 acting on Histone H3 lysine K4 (H3K4) methylation, related to the transcription of HES1. In other words, lncRNA PAUPAR reduces HES1 transcription, which reduces proliferation and invasion [73].

Another notable lncRNA is NUMB. It is encoded upstream of the NUMB protein gene, which can reduce tumor formation and prevent invasion in UM cell lines. Hypermethylated in cancer 1 (HIC1) regulates lncRNA NUMB in uveal melanoma. HIC1 promotes lncRNA expression, acting as a transcriptional activator [74]. LncRNA NUMB acts as a tumor suppressor by inhibiting cell proliferation and invasion. However it is downregulated in UM [74,83].

LncRNA Calcium Activated Nucleotidase 1 (CANT1), also known as CASC15-NT1, is a typical cancer-associated lncRNA. This downregulated tumor suppressor lncRNA is an isoform of lncRNA Cancer Susceptibility 15 (CASC15) implicated in UM. [75,84]. LncRNA CANT1 controls the expression of the lncRNA X-inactive specific transcript (XIST) (XIST is implied in chromosome X gene repression) [85]. Moreover, lncRNA CANT1 regulates JPX or five prime to Xist (FTX) transcription factors through their promoter methylation by H3K4. This pathway contributes to tumorigenesis in UM [75].

The lncRNA ZNF706 Neighboring Transcript 1 (ZNNT1) promotes autophagy in UM through mTOR inhibition but is downregulated in tumors. LncRNA ZNNT1 controls the expression of autophagy-related 12 (ATG12) and modulates the ATG12-ATG15 conjunction. This mechanism produces cell death and tumorigenesis suppression. Furthermore, the lncRNA could act in proteosome inhibitor-mediated apoptosis [76].

It is worth highlighting that several lncRNAs play dual roles such as the lncRNA Small Nucleolar RNA Host Gene 7 (SNHG7), which has tumor suppressor or oncogenic activity depending on the kind of cancer. For instance, it has oncogenic properties in several cancers such as pancreatic, bladder, colorectal, gastric, and breast cancer [77,86,87,88,89,90]. Whereas, in UM, lncRNA SNHG7 can inhibit malignant transformation due to its effect on the Enhancer of Zeste 2 Polycomb Repressive Complex 2 Subunit (EZH2) protein, which regulates cell proliferation, the cell cycle, and apoptosis. Specifically, the lncRNA SNHG7 can inhibit EZH2, reducing UM progression [77]. Similarly, it has been described that in lung cancer, this lncRNA can also work as a sponge of the oncogenic miR-181 (which promotes cell proliferation and migration), reducing the activity of miR-181 and, therefore, the tumoral progression [91].

The lncRNA Growth Arrest Specific 5 (GAS5) is downregulated in UM, and is usually associated with a bad prognosis. This lncRNA blocks the oncogenic miR-21, which leads to epithelial-mesenchymal transition (EMT) via Phosphatase and tensin homolog (PTEN) activation. Therefore, the final result of lncRNA GAS5 downregulation is invasion [78].

### 3.2. Oncogenic LncRNAs in Uveal Melanoma

Some examples of oncogenic lncRNAs in UM are presented below (Figure 3). As in the tumor suppressor case, oncogenic lncRNA could activate oncogenic molecules or inhibit tumor suppressor elements.

A classic lncRNA with an oncogenic role is lncRNA Metastasis Associated Lung Adenocarcinoma Transcript 1 (MALAT1) (Figure 3). It is also known as nuclear-enriched abundant transcript 2 (NEAT2), and is upregulated in different kinds of cancer such as lung, glioma, bladder, pancreatic, gastric, colorectal cancer, and UM. The overexpression of this lncRNA is associated with low survival rates [92]. It is related to cell cycle progression and proliferative phenotypes due to E2R1 and p53 regulation. MALAT1 depletion makes tumor cells sensitive to p53. Moreover, lncRNA MALAT1 interacts with splicing factors to affect the alternative splicing of some mRNAs [93]. According to some UM studies, lncRNA MALAT1 reduces the expression of miR-140, which promotes proliferation and invasion in UM [64]. Other studies suggest that lncRNA MALAT1 upregulates Homeobox C4 (HOXC4), a HOX family member, by inhibiting miR-608, which also promotes UM tumorigenesis [65]

The oncogenic lncRNA Plasmacytoma variant translocation 1 (PVT1) acts as a miRNA sponge in several cancers, leading to enhanced proliferation and metastasis [94]. For instance, lncRNA PVT1 traps miR-186 in gastric cancer [95], miR-26b in melanomas [96], miR-448 in pancreatic cancer [97], and miR-203 in esophageal carcinomas [98]. Furthermore, in UM, lncRNA PVT1 downregulates the expression of miR-17-3p, increasing metastasis. This process is caused by oncogenic murine double minute 2 (M2M2) expression and the decrease of p53 tumor suppressor activity [67]. Other authors have reported that lncRNA PVT1 positively regulates the expression of EZH2 in UM cell lines, which leads to carcinogenic effects and poor prognosis [66].

Another oncogenic lncRNA is lncRNA Rhophilin Rho GTPase binding protein 1 antisense RNA 1 (RHPN1-AS1), which is overexpressed in UM compared to healthy tissues. This lncRNA is associated with angiogenesis, cell adhesion, and extracellular matrix organization. Therefore, lncRNA RHPN1-AS is implicated in the epithelial-mesenchymal transition (EMT) through the Transforming growth factor beta (TGF-β) signaling pathway interaction [68]. In other cancers, it acts with/against miRNAs to promote cancer such as in cervical cancer through the miR-299-3p/Fibroblast Growth Factor 2 (FGF2) axis [99], in glioma targeting miR-652-5p/Regenerating islet-derived protein 3-alpha (REG3A) [100], in breast cancer sponging miR-6884-5p [101], or sponging miR-7-5p in colorectal cancer [68], but none of these relations have been studied in UM yet.

The lncRNA Homeobox A11 (HOXA11), also known as NCRNA00076, belongs to the Homeobox A (HOXA) cluster with lncRNA Homeobox A10 antisense (HOXA10AS) and HOXA Distal Transcript Antisense RNA (HOTTIP), all of which are involved in cancer proliferation, invasion, migration, and chemoresistance [102,103]. LncRNA HOXA11AS is upregulated in many cancers such as non-small cell lung cancers, osteosarcoma, glioma, hepatocellular carcinoma, gastric, breast, cervical cancer, and UM [104]. LncRNA HOXA11 acts as a sponge for the tumor suppressor miR-124 in breast cancer and, through interaction with EZH2, inhibits protein p21 (p21) [105]. In UM, the high concentration of lncRNA HOXA11AS leads to proliferation and invasion. This effect could be reversed with miR-124 mimic transfection [69].

The high levels of lncRNA Ferritin Heavy Chain 1 Pseudogene 3 (FTH1P3) in UM correlate with proliferation, cell cycle, and migration. It is dysregulated in other cancers such as lung, cervical, glioma, esophageal carcinoma, and breast cancer [106,107,108,109,110]. It is suggested that lncRNA FTH1P3 decreases miR-224-5p expression and enhances Fizzled 5 and Ras-related C3 botulinum toxin substrate 1 (Rac1) expression, promoting cell cycle progression and migration [70].

Purinergic Receptor P2X 7 (P2RX7), a ligand-gated ion channel receptor, is overexpressed in several cancers and is expressed differently in many tissues. This receptor participates in tumor growth, differentiation, metabolism, migration invasion, and cell death, so the P2RX7 overexpression is related to poor prognosis in patients [111]. It has been reported that the lncRNA P2RX7-V3 variant acts as an oncogene because of the positive correlation with the P2RX7 receptor. Although the action mechanism is not clear, the lncRNA P2RX7-V3 variant is upregulated, and is involved in the tumor maintenance of UM cell lines. Moreover, the analysis of the P2RX7-V3 lncRNA targets demonstrates that the lncRNA participates in the Phosphatidylinositol 3-kinase (P13K)-Protein kinase B (AKT) pathway promoting tumorigenesis [63].

The lncRNA LINC00518 is overexpressed in the cytoplasm of UM cells, but it has not been detected in extracellular fluids. Therefore, it is currently not useful as a biomarker. It is proposed that lncRNA LINC00518 could act as a miRNA sponge modulating the metastatic process; however, the cellular pathway has not been well established yet [71].

The lncRNA LOC100132707 is overexpressed in metastatic UM, and is correlated with the Janus kinase 2 (JAK2)/Signal Transducer and Activator of Transcription 3 (STAT3) pathway, which are signal transducers and activators of transcription. The activation of this signaling leads to migration and invasion [72].

## 4. LncRNAs as Therapeutic Agents

Selecting a proper lncRNA for therapeutic use requires tissues to present different expression profiles between tumors and healthy cells. It is essential that the differential levels are due to aberrant cancer expression to target the tumor exclusively. If the therapy is effective (upregulating tumor suppressor lncRNAs or downregulating oncogenic ones), the modulation of lncRNA expression should induce cell death, or reduction in tumor size or motility [112].

Several features make lncRNAs ideal molecules for cancer treatment: (a) lncRNA concentration is lower than mRNAs, besides lncRNA have a fast turnover, meaning they react fast with the targets; (b) lncRNAs are specific for certain cells/tissues specific, making them ideal for the selection of cell subpopulations and develop selective therapeutics; (c) LncRNAs can control chromatin modifications; therefore, targeting lncRNAs could be exploited to modulate the epigenetics of the cells; (d) LncRNAs could modulate chromatin function, regulate the assembly of nuclear bodies, or affect stability and translation of mRNA; (e) LncRNAs have many binding sites, thus, blocking different lncRNAs domains could lead to more efficient therapy because of their effect in multiple proteins of dysregulated pathways; and (f) targeting lncRNAs provides simultaneous effects in several pathways, so there are fewer chances to develop tumor resistance [8,112,113,114,115].

There are several strategies to modulate upregulated lncRNA expression depending on their subcellular localization and mechanism of action [31,41,50,116,117,118]:

- Inhibit oncogenic lncRNA expression using specific siRNAs, antisense oligonucleotides (ASOs), gapmers, ribozymes, and Dnazymes, synthetic lncRNA mimics, or CRISPR systems.
- Block the interaction between lncRNAs and their target molecules (e.g., regulatory factors or promoters) or affect the lncRNA secondary structure with aptamers or small synthetic molecules.

In the case of downregulated lncRNAs, therapies are focused on increasing tumor suppressor lncRNA levels to restore the normal expression levels [119].

Although new therapies against lncRNAs have been developed, there are many challenges in delivering oligonucleotides because of their natural degradation, immune system activation, and difficulties in targeting cancer cells and their organelles [120]. Various delivery systems have been developed to overcome these limitations such as liposomes, micelles, dendrimers, inorganic particles, carbon nanotubes, nanoparticles, viral nanocarriers, polymeric or peptide nanoparticles, metallic nanoparticles, and others [116,117,118,121,122,123,124,125,126]. Through the use of delivery systems, side effects can be reduced due to accurately targeting the tumoral cells (e.g., using polycation gene vectors for delivering lncRNA Maternally Expressed 3 (MEG3) in hepatocellular carcinoma [127], or gold nanoparticles with Tyrosine Aminotransferase (TAT) peptide to deliver specifically into lung cancer cells ASOs against lncRNA MALAT) [128]. Notably, by using nanoparticle-based approaches, stimuli-sensitive systems can be implemented to improve control over the release. One example of this is the delivery of siRNAs against the lncRNA Differentiation Antagonizing Non-Protein Coding RNA (DANCR) with pH-sensitive amino lipid, polyethylene glycol, and peptide formulations. These formulations are being used to treat triple-negative resistant breast cancer models [129]. Moreover, exosomes are gaining relevance in this field because of their low immunogenicity and good biocompatibility and stability. However, exosomes are not as tunable as other nanoformulations. For this reason, they are used to hybridize with other nanoparticles (e.g., liposome-exosome nanoformulations) to deliver CRISPR-Cas 9 [130].

Regarding UM, therapies based on lncRNA could target tumor melanocytes by taking advantage of lncRNA tissue specificity and delivery systems. Selecting the proper oncogenic lncRNA and delivering therapies against it would reduce tumor size, avoiding side effects. Moreover, these therapies could reach metastatic cells such as liver metastasis (e.g., targeting lncRNA Receptor Tyrosine Kinase Like Orphan Receptor 1 antisense 1 (ROR1-AS1) or lncRNA Homeobox D antisense (HOXD-AS1)) [131,132], which is the main cause of death in UM patients.

## 5. LncRNAs as Diagnostic Agents

It is well known that early diagnosis is fundamental to improve the survival of cancer patients. In addition, it is also necessary to monitor cancer progression. In this regard, X-ray, magnetic resonance imaging, histopathology, molecular pathology, circulating tumor cell detection, and tomography are the most common diagnostic methods in the clinic, but most of them are expensive and invasive techniques; therefore, new non-invasive, real-time, and reproducible diagnostics are desired [133,134].

UM used to be diagnosed by enhanced depth imaging optical coherence tomography (EDI-OCT) and fluorescein or indocyanine green angiography (FFA or ICG) [50], however, metastatic UM has a challenging detection because of the early dissemination and micrometastasis, principally in the liver [135]. Thus, UM is usually detected after the tumor has grown significantly, affecting liver function [136]. Cytogenetic diagnosis is recommended, but these kinds of tests are invasive and conducted after tumor biopsies [135]. For liver metastasis, the standard techniques are abdominal ultrasound and liver biochemical function test. In general, blood biomarkers are the best option to diagnose, establish a prognosis, and predict therapeutic response in metastatic and non-metastatic UM [135].

Many lncRNAs are cancer-specific or aberrantly expressed in some cancer tissues, making them exploitable as biomarkers. LncRNAs could indicate the presence or absence of cancer and even the disease progression. Furthermore, lncRNAs are stable in blood, saliva, and urine, and they are detected in circulating extracellular vesicles [137]. Therefore, they can be excellent markers in non-invasive tests for personal and precision oncology [50,138,139,140].

Interestingly, as stated before, lncRNA expression could be correlated to the tumor stage and, therefore, with cancer prognosis or tumor recurrence. This correlation is explained because different types of tumors at various stages of progression present distinct levels of lncRNA depending on the cellular pathways affected [133].

Some lncRNAs are already being used as biomarkers in the clinic such as the lncRNA Prostate cancer antigen 3 (PCA3) approved by the FDA as a prostate cancer biomarker, with a sensitivity of 58–82% and a 56–76% specificity [141]. In UM, several lncRNAs are already used for diagnosis in basic research (Table 3) [133].

The current lncRNA detection methods include northern blot, qRT-PCR, RNA-seq, and microarrays. Although it is possible to detect lncRNA with these techniques, not many lncRNA biomarkers are approved for routine diagnoses in humans. The reason for this is the lack of extensive cohort studies and inconsistent acquisition and analysis methods. Furthermore, there is no agreement on suitable sample tissue, RNA isolation method, sequencing, analysis, or biostatistics techniques; all these steps should be standardized for proper clinical translation.

Nowadays, some blood biomarkers are used in UM such as tumor-associated antigen (MIA), osteopontin, and S-100β, among other hepatic markers. These biomarkers are present at high concentrations in UM patients with liver metastasis [136]. However, any lncRNA biomarker currently used in the clinic has an enormous potential to be applied for UM detection and monitoring, because they would be found in blood and are cancer-specific, sensitive, and the technique is cost-effective, rapid, and non-invasive [135].

In summary, using two or more lncRNAs in combination with currently used biomarkers significantly increases the specificity and sensitivity in cancer diagnosis [94,95].

## 6. LncRNA-microRNA Interactions Related to Uveal Melanoma

As previously mentioned, the interaction between different ncRNAs permits regulatory subcategories in the genome, which means that ncRNA levels depend on other ncRNAs. In particular, the relationship between lncRNAs and microRNAs is very important due to the crucial roles in both the homeostasis and disease processes [5].

Despite the large number of lncRNA–microRNA interactions in nature, very few have been described in UM. This absence is related not only to the lack of research in UM, as it is a rare disease, but also because of the novelty of this research field. Recently, a database called VECTOR (uVeal mElanoma Correlation NeTwORk) has been published to predict RNA interactions in UM [142], which will be very helpful in this field.

The main contributions in this area are discussed below.

LncRNA PVT1 is an oncogenic lncRNA related to metastasis risk. This lncRNA binds miR-17-3p, reducing its expression levels. Moreover, miR-17-3p often downregulates MDM2 expression, a protein that inhibits p53. In UM, the lncRNA PVT1 is highly expressed, and therefore miR-17-3p is inhibited, leading to MDM2 upregulation and p53 inhibition [67]. This relationship has been described in other cancers such as gastric cancer or neuroblastoma [138,143]. The study of this regulatory pathway in which lncRNAs and microRNAs are involved might allow for the design of promising therapies to reduce the expression of lncRNA PVT1 or increase the levels of miR-17-3p to achieve cancer regression. Moreover, the presence of the high levels of lncRNA PVT1 could be used as a biomarker to detect UM [67].

Another well-known oncogenic lncRNA is MALAT1. This lncRNA is also implicated in metastasis and correlates with advanced tumor stages and poor survival in several cancers such as lung adenocarcinoma, breast cancer, hepatocellular carcinoma, gastric cancer, pancreatic cancer, and others [144]. LncRNA MALAT1 regulates miR-608, a tumor suppressor that inhibits HOXC4, a homeobox family’s transcription factor [139], and AKT2, an oncogene kinase [140], leading to apoptosis. In UM, MALAT1 is overexpressed, which reduces the expression of miR-608, leading to an increase of HOXC4. Thus, these processes together enhance UM cell proliferation and invasion [65].

LncRNA HOXA11AS is an oncogenic lncRNA involved in UM progression, and its overexpression is related to cell growth, migration, and apoptosis evasion. This lncRNA can bind EZH2 [69], a polycomb family member with a key role in the cell cycle, cell death, and cell lineage determination [145]. Additionally, lncRNA HOXA11AS works as a miR-124 sponge, which controls EZH2 expression levels and causes apoptosis and/or autophagy [146]. Therefore, low miR-124, mediated by HOXA11AS, releases EZH2, inhibiting the tumor suppressor p21. Interestingly, it has been shown that by increasing miR-124 levels, proliferation and invasion were reduced in UM cells [69], highlighting the potential use of lncRNAs as therapeutic targets.

## 7. Conclusions

LncRNA are fascinating molecules involved in UM progression. Regarding their biological implications, lncRNA can interact with diverse types of molecules. For instance, they can interact with proteins acting as protein scaffolds, oligonucleotide decoys, or guides; also with DNA, acting as guides or enhancers; or with other RNA structures as miRNA sponges or mRNAs inhibitors.

Since LncRNAs are implicated in tumor formation and progression in uveal melanoma, they can be excellent biomarker candidates for non-invasive diagnostic techniques. In this scenario, oncogenic lncRNAs such as PVT1, CASC15, or MALAT1 are perfect candidates to develop diagnostic methods because they are more abundant in UM tumors or even patient serum. Furthermore, oncogenic lncRNAs are also remarkable targets to inhibit tumors, increase drug sensibility, and prevent chemoresistance or future relapses. In particular, tumor suppressor lncRNAs such as lncRNA PAUPAR or NUMB can be used as therapeutic molecules to reduce tumor progression in UM. It is worth mentioning that these therapeutic approaches face some challenges associated with drug delivery in vivo (e.g., stability, internalization), which might be overcome through the use of modern nanocarriers.

Finally, it is clear that a better understanding the lncRNAs’ roles will provide us with new tools to detect and treat UM, which are needed to tackle this terrible disease. In this regard, this review could be very valuable to better understand the implications of LncRNAs in UM and promote this area of research.

## Figures and Tables

**Table 1:** Biological processes and examples of lncRNAs.

Biological Process	LncRNA	Citation
Transcription	lncRNA NRON, lncRNA HSR1	[10,11]
Splicing	lncRNA MALAT, lncRNA ASCO	[12,13]
Translation	lncRNA HULC	[14]
RNA localization	lncRNA XIST	[15]
RNA decay	lncRNA gadd7	[16]
RNA editing	lncRNA CTN	[17]
Epigenetic remodeling	lncRNA HOTAIR	[18]
Genome integrity	lncRNA NORAD, lncRNA CONCR	[19,20]
Structural functions	lncRNA NEAT1, lncRNA FIRRE	[21]
Cellular organelle functions	lncRNA RMRP, lncRNA SAMMSON	[22,23]

**Table 2:** LncRNAs implicated in UM progression.

LncRNA Name	Mechanism	Role	Reference
lncRNA CASC15	Switches tumor phenotype through MITF and/or SOX10	Oncogenic	[56]
lncRNA P2RX7-V3	Affects PI3K/AKT pathway	Oncogenic	[63]
lncRNA MALAT1	Suppresses miR-608. Promotes miR-140 expression and suppresses Slug and ADAM10 expression.	Oncogenic	[64,65]
lncRNA PVT1	Regulates the expression of EZH2 and blocks miR-17-3p	Oncogenic	[66,67]
lncRNA RHPN1-AS1	Participates in TGF-β pathway	Oncogenic	[68]
lncRNA HOXA11-AS	Suppresses p21 and acts as a sponge of miRNA -124	Oncogenic	[69]
lncRNA FTH1P3	Suppresses miR-NA 224-5p expression and promotes the expression of Rac1 and Fizzled 5	Oncogenic	[70]
lncRNA LINC00518	Participates in the metastatic process	Oncogenic	[71]
lncRNA LOC100132707	Promotes migration via JAK2	Oncogenic	[72]
lncRNA PAUPAR	Modulates HES1 expression	Tumor suppressor	[73]
lncRNA NUMB	Restores the expression of HIC1 (Hypermethylated in cancer 1)	Tumor suppressor	[74]
lncRNA CANT1	Modulates JPX or FTX by methylation at their promoters	Tumor suppressor	[75]
lncRNA ZNNT1	Promotes autophagy	Tumor suppressor	[76]
lncRNA SNHG7	Regulates EZH2 pathway	Tumor suppressor	[77]
lncRNA GAS5	Induces PTEN expression	Tumor suppressor	[78]

**Table 3:** UM lncRNA diagnosis candidates.

LncRNA	Expression Level	Source	Association	Reference
lncRNA PVT1	Up	Tumor, gastric juice, serum	Poor overall survival	[94]
lncRNA HOXA11AS	Up	Tumor	Poor overall survival	[104]
lncRNA SNHG7	Down	Tumor	Higher tumor-node-metastasis stage (TNM) and poor histological type	[77,79,97]
lncRNA MALAT1	Up	Tumor, urine, serum	Melanoma progression and metastasis	[133]
lncRNA CASC15	Up	Tumor	Cancer recurrence	[133]
